# The clinical application of Chinese herbal medication to depression: A narrative review

**DOI:** 10.3389/fpubh.2023.1120683

**Published:** 2023-03-09

**Authors:** Dongyu Kang, Huixi Dong, Yidong Shen, Jianjun Ou, Jingping Zhao

**Affiliations:** ^1^Department of Psychiatry and Mental Health Institute of the Second Xiangya Hospital, Central South University, The China National Clinical Research Center for Mental Health Disorders, National Technology Institute of Psychiatry, Key Laboratory of Psychiatry and Mental Health of Hunan Province, Changsha, Hunan, China; ^2^Mental Health Center of Xiangya Hospital, Central South University, Changsha, Hunan, China

**Keywords:** depression, Chinese herbal medicine, pharmacological therapy, efficiency, safety, physical comorbidity

## Abstract

Depression severely impairs psychosocial functioning and quality of life, which places a huge burden on patients and their families. However, the physiological mechanism of depression remains unknown. Treatment with existing antidepressant medications is effective in around 50% of patients according to various studies, but is associated with severe side effects including nausea and headaches. Chinese herbal medicine (CHM) has been approved and widely used for depression as an alternative medicine in Chinese culture for decades. It has certain advantages and potential in the prevention and treatment of depression. In this review, we summarize the currently available evidence for the efficacy of CHM for the treatment of depression and physiological diseases comorbid with depression. We further discuss the possible mechanisms of action of CHM and the relationships to our current understanding of depression. The majority of current evidence has suggested that the combined treatment with CHM and mainstream antidepressants improves the response rate and reduces the side effects, while CHM alone could be more effective than placebo. However, the results should be carefully interpreted due to the shortcomings of existing clinical trials and a high risk of bias in meta-analyses. Our review provides a summary of the current applications and understanding of widely used CHMs for depression.

## Introduction

Major depressive disorder (MDD) is a common illness that severely impairs psychosocial functioning and quality of life; it is currently the third commonest cause of burden of disease worldwide, with expectations that it will become the commonest by 2030 (1). A recent, large-scale cross-sectional epidemiological study suggested that depressive disorders are the most prevalent mood disorders in China, with a lifetime prevalence of 6.8%, and that among the depressive disorders, MDD has the highest prevalence with 3.4% (2). According to the current diagnostic systems, MDD is mainly diagnosed through the presence of several key symptoms, but most of these symptoms are not specific to MDD (1) and may be present in many physical diseases or other conditions. If we take the presence of any depressive symptoms into account, the incidence may be much higher.

The pathophysiology of depression (or depressive symptoms) remains to be fully elucidated, which poses challenges to its treatment and prevention. The neurotransmitter hypothesis has long been welcomed as the potential mechanism underlying MDD, and most of the antidepressants used clinically were studied and developed based on this hypothesis. In the mid-twentieth century, the anti-hypertensive reserpine was found to be able to reduce monoamine levels and trigger major depression, which increased interest in the potential role of monoamine neurotransmitters in the pathology of MDD. Subsequently, tricyclic antidepressants and monoamine oxidase inhibitors have been used to relieve depression by enhancing monoamine neurotransmission, an effect that supports the monoamine theory of MDD (3). However, this model does not explain why antidepressants take several weeks to take effect (4). Considering that depression is closely related to trauma and life events, the corresponding physiological mechanisms have been studied. In addition to neurotransmitter theories, the hypothalamic-pituitary-adrenal (HPA) axis is a key component of a comprehensive neurobiological model explaining the long-lasting consequences of early life trauma, as studies have shown increased activity of the HPA axis in animal stress models (5). This hypothesis is supported by clinical studies indicating that physical abuse in childhood can result in enhanced activity of the HPA axis when the same individuals are subsequently exposed to standardized psychosocial stressors as adults (6). Another pathophysiological hypothesis relating to MDD involves immune dysfunction and neurotrophic factor, which may become a potential treatment target for MDD (7). Individuals with MDD show increased inflammatory system activation, which is under the inhibitory physiological control of cortisol. As an important component of the physiological stress-sensing and reactive pathways, the immune system may also have a role in MDD. Several animal studies have suggested that peripheral immune dysfunction and neuroimmunology are involved in the mechanisms of MDD (8).

The Chinese traditional medicine's theory of depression emphasizes an individualized diagnostic and treatment system based on each patient and the environment the patient encounters. The liver has been considered as the viscera governing emotions and the free-coursing of qi and blood. A weakened liver function may lead to the disturbance and obstruction of qi and blood and cause depression. Qi is the basic element in Chinese traditional medicine, which flows within the body and formed everything, and maintains all functional activities of an organic body. A qi deficiency may lead to a lack of energy, a common symptom of depression. The balance of yin and yang, originating in ancient Chinese philosophy, was also considered in the traditional Chinese theory of medicine. A depression could be caused by a disturbance of yin or yang, especially by the deficiency of yin. This unique view of depression may hardly correspond with the current DSM or ICD definitions but guides the application of Chinese traditional herbal medicine in treating depression (9).

In the Canadian Network for Mood and Anxiety Treatments (CANMAT) clinical guidelines for the management of adults with MDD, selective serotonin reuptake inhibitors (SSRIs), serotonin and norepinephrine reuptake inhibitors (SNRIs), agomelatine, bupropion, and mirtazapine are recommended as first-line treatments (10). However, a real-world study on antidepressants suggested that 25% of the side effects were “very bothersome” or worse, while 40% of the patients in the study mentioned the side effects to their physicians (11). The main adverse events of antidepressants include nausea/vomiting, extrapyramidal effects, weight gain, and headache, which significantly influence a patient's quality of life and compliance with medication (12). Complementary and alternative medicine is becoming more popular globally (13). About 16–44% of people with mental illnesses use complementary and alternative medicines (14), and a significant majority of them suffer from depression (15).

Chinese herbal medicine (CHM) is the most popular complementary and alternative medicine in Chinese culture (16). A large number of people believe that CHM has certain advantages and potential in the prevention and treatment of diseases, because of the years of practical experience with it (17). Some studies have shown that CHM monotherapy is superior to placebos (18), and that, compared to antidepressants alone, the combination treatment of CHM and first-line antidepressants can significantly improve both response and remission rates and reduce the side effects (19). Most CHMs are derived from natural products and composed of complex formula. Based potentially on the Chinese traditional concepts of “Balance” and “Natural,” many Chinese people believe that CHMs have advantages over modern Western medicine in terms of side effects. However, a number of studies have noted the lack of research on their potential side effects. Toxicity may potentially arise both intrinsically, through the natural compounds present in the herbs, and extrinsically, as a result of contaminants present during the cultivation, harvesting, or processing of the herbs (20). Therefore, a range of adverse effects may be induced by CHM use, including liver (21) and kidney dysfunction (22, 23), allergic reactions (24), and others [e.g., bleeding (25)]. Thus, it is important to exercise caution when prescribing CHMs, and take the quality, dosage, duration, and related interactions of their use into consideration to further minimize adverse effects.

Some previous studies have indicated that CHM appears to be associated with less adverse events while being as effective as antidepressants (26); meanwhile, others have indicated that there is insufficient evidence to support its effect on depressive episodes, which may be due to the low quality of evidence arising from existing trials (27). Hence, to better understand the efficacy and safety of each CHM for depression, we conducted this narrative review. In this review, we focused on summarizing current research on depression with and without physical comorbidities.

## Efficiency and safety of CHM for MDD

Chinese herbal medicine is a major form of complementary and alternative medicine in China (28), an increasing industry, and has rapidly developed over the past several decades. Several clinical studies and a meta-analysis have indicated that CHM has therapeutic effects on depression with fewer side effects than first-line antidepressants (19). The result of a large meta-analysis supported the idea that CHM is more effective than placebos and as effective as antidepressants in the treatment of depression; however, the poor quality of the included randomized controlled trials (RCTs) and the lack of adequate safety data made it difficult to draw a definite conclusion (26). Butler and Pilkington conducted a review on the use of CHM for MDD, which indicated that almost all the included clinical trials favored its use, with no significant differences when comparing CHM to current medication. Greater effects were observed for CHM compared to placebo, and reduced adverse events rates were observed when CHM was used as additive therapy (29). In another systematic review of RCTs, when compared to fluoxetine, CHM displayed a similar effect on relieving depression according to Hamilton Depression Rating Scale (HAMD) assessment with fewer adverse events; however, due to the poor quality of the included trials and the potential publication bias of the review, the authors noted that no definite conclusion should be drawn regarding the effect and safety of CHM for depression when compared to fluoxetine (30). A recent meta-analysis of RCTs conducted by Wang et al., including 40 studies with Cochrane Risk of Bias scores higher than 4, showed that CHM monotherapy had better clinical effects than placebos and was as effective as conventional Western medications. Western medication was more effective when combined with CHM, with fewer adverse events, but the quality of the relevant studies was not sufficient to draw any definite conclusions (31).

Although the diagnosis of depression was only introduced to China during the last century, the description of depression-like symptoms has been reported in ancient Chinese medicine books since 400–300 B.C. The “Yellow Emperor's Canon of Medicine,” one of the earliest texts on the theory and practice of traditional Chinese medicine, suggested that “…heart is the organ similar to the monarch and is responsible for spirit and mental activity…” and divided “melancholy” into five different types. In “Treatise on Cold Pathogenic and Miscellaneous Diseases,” the Ganmai Dazao decoction was introduced to treat mood-related conditions. Over time, about 100 different Chinese herbal formulas have been developed and used to treat depression in China, among which a great variety exists due to the complexity of traditional Chinese medicine diagnoses and practitioners' personal experiences. Xiao Yao decoction is the most frequently used formula, followed by Chaihu Shugan decoction and Ganmai Dazao decoction. We will, respectively, summarize the existing published clinical studies and reviews for the four most frequently used CHMs for depression.

### Xiao Yao San

As the most frequently used CHM, Xiao Yao San decoction and its modifications have been used for the treatment of depression in China for centuries (26). It usually contains eight commonly used herbs: BaiShao (*Paeonia lactiflora* Pall.), ChaiHu (*Bupleurum chinense* DC), BaiZhu (rhizome of *Atractylodes macrocephala* Koidz.), DangGui (*Angelica sinensis* [Oliv.] Diels), GanJiang (*Zingiber officinale* Rosc.), FuLing (*Pori cocos* [Schw.] Wolf.), GanCao (*Glycyrrhiza uralensis* Fisch.), and BoHe (*Mentha haplocalyx* Briq.). The prescriptions based on Xiao Yao San can be administered as pills, powder, or decoction. Zhang et al. conducted a systematic review of RCTs on Xiao Yao San for treatment of depression in 2012, which included 26 trials for analysis. The results indicated no significant difference between Xiao Yao San and antidepressants in overall effectiveness (risk ratio 1.05 [1.00, 1.11]; *P* = 0.07), or in HAMD score decrease (0.43 [−2.14, 2.99]; *P* = 0.74). The meta-analysis of four trials significantly favored Xiao Yao powder compared to antidepressants (−3.97 [−5.52, −2.41]; *P* < 0.001) for reducing Self-Rating Anxiety Scale (SAS) scores. These results support Xiao Yao San in combination with antidepressants vs. antidepressants alone, whether in terms of the overall effects or the effects on HAMD or SAS scores. However, these effects were mainly due to one study alone, as the remaining three studies included in the meta-analysis showed no significant differences. The combination of Xiao Yao San and antidepressants also resulted in fewer side effects than antidepressants alone. Nevertheless, this result should be carefully interpreted due to concerns about publication bias and poor clinical research quality (32). The potential mechanisms of Xiao Yan Wan could involve the modulation of monoamine neurotransmitters, and regulation of the HPA axis, but more solid research is required (33).

### Shuganjieyu capsules

Shuganjieyu was the first CHM approved for the treatment of depression by China's drug regulatory agency in 2008, and is composed of GuanYeLianQiao (*Hypericum perforatum* L.) and CiWuJia (*Acanthopanax senticosus* [Rupr. Maxim.]). Zhang et al. conducted a systematic review in 2014 which included two studies comparing Shuganjieyu capsules with placebos and five studies comparing the combination of Shuganjieyu and venlafaxine with venlafaxine alone. The results supported the use of Shuganjieyu capsules over placebos [95% confidence interval (CI) = 5.61–2.73, *P* < 0.001]; however, no significant difference was found between the combination of Shuganjieyu and venlafaxine with venlafaxine alone. The combination of Shuganjieyu and venlafaxine resulted in fewer side effects (34). One randomized controlled study evaluated the effect of Shuganjieyu capsules on generalized anxiety disorder, demonstrating an effect on both anxiety symptoms and combined depressive symptoms (35). In a rat model of depression, Shuganjieyu capsules increased phosphorylation levels of the phosphorylation cyclic adenosine monophosphate response element binding protein and brain-derived neurotrophic factor (BDNF) expression in the medial prefrontal cortex and hippocampal CA3 area, which is believed to be the major mechanism of its antidepressant effect (36).

### Chaihu Shugan San

Chaihu Shugan San was noted in the Chinese medical classic “Jingyue Quanshu” in the year 1640 as an efficient treatment for depression via relieving liber-qi stagnation caused by anger or distress, according to its theory. Chaihu Shugan San, although bearing a similar name to the Shuganjieyu capsule, consists of seven different Chinese herbs: CaiHu (*Bupleurum chinense* DC.), ChenPi (*Citrus reticulata* Blanco), BaiShao (*Paeonia lactiflora* Pall.), ZhiKe (*Citrus aurantium* L.), XiangFu (*Cyperus rotundus* L.), ChuanXiong (*Ligusticum chuanxiong* Hort.), and GanCao (*Glycyrrhiza uralensis* Fisch.). Wang et al. conducted a meta-analysis of the clinical effectiveness of Chaihu Shugan San in depression in 2012, which included 10 studies with 835 patients with depression. In this meta-analysis, Chaihu Shugan San demonstrated a significant efficacy in decreasing HAMD scores. When combined with antidepressants, it was found to be more effective than antidepressants alone (95% CI = −5.09 to −2.03; *P* < 0.001), with higher effectiveness [odds ratio (OR) = 3.31 95% CI = 1.80–6.10; *P* < 0.001] and recovery (OR = 2.32, 95% CI = 1.61–3.34; *P* < 0.001) rates. Two further studies evaluated Chaihu Shugan San as a monotherapy for depression compared with placebo, and both favored it in terms of HAMD scores but not for the effectiveness nor recovery rates (37). A proteomics study suggested that its antidepressant effect could involve multiple targets and pathways, which may include regulations of 110 DEPs and neurotransmitter transmission cycles (38). Besides, the PI3K/AKT pathway could be a potential mechanism and quercetin, luteolin, and kaempferol were probable active compounds (39). To provide a concrete conclusion, further full-scale, well-designed, and timely registered clinical trials are necessary in the future.

### Ganmai Dazao decoction

Ganmai Dazao decoction was first documented in Jin Gui Yao Lue, first published in the year 1066 by Zhang Zhongjing. Its main ingredients include GanCao (*Glycyrrhiza uralensis* Fisch.), XiaoMai (*Triticum aestivum* L.), and DaZao (*Ziziphus jujube* Mill.) in a ratio of 3:5:5. Today, it is one of the most commonly used herbal formulas for depression in China. In 2014, Yeung et al. published a meta-analysis of the efficacy and safety of Ganmai Dazao decoction for depression. The results indicated that it was comparable to antidepressants in terms of the effects on the HAMD score. There was no significant difference in effectiveness rates when comparing Ganmai Dazao plus antidepressants with antidepressants alone. Meanwhile, adverse events were more common with antidepressants. This review suggested that Ganmai Dazao has the potential as an antidepressant agent with few side effects. However, like other research into CHM, the clinical research involved was all published in Chinese and had poor study quality (40). Another systematic review and meta-analysis of Ganmai Dazao decoction failed to provide evidence for its superiority over antidepressants for depression, partly because of the controversial results and high risk of bias of the included clinical trials (41). One animal study showed that its action might be modulated by 5-HT1a and GABA receptors (42).

Table 1 summarizes the components and possible mechanisms of the above four Chinese herbal medicines for depression.

**Table 1:** The components and possible mechanisms on depression of four Chinese herbal medicines.

	**Components**	**Possible antidepressant mechanism (modern medicine theory)**	**Possible antidepressant mechanism (traditional Chinese medicine theory)**
Xiao Yao San	BaiShao (*Paeonia lactiflora* Pall.), ChaiHu (*Bupleurum chinense* DC), BaiZhu (rhizome of *Atractylodes macrocephala* Koidz.), DangGui (*Angelica sinensis* [Oliv.] Diels), GanJiang (*Zingiber officinale* Rosc.), FuLing (*Pori cocos* [Schw.] Wolf.), GanCao (*Glycyrrhiza uralensis* Fisch.), BoHe (*Mentha haplocalyx* Briq.).	Increase 5-HT and DA levels, inhibit hyperactivity of the HPA axis, and upregulate BDNF.	Harmonize the liver and spleen, sooth liver and relieve depression, nourish blood, and invigorate the spleen.
Shuganjieyu capsules	GuanYeLianQiao (*Hypericum perforatum* L.), CiWuJia (*Acanthopanax senticosus* [Rupr. Maxim.]).	Increase 5-HT, NE, and DA levels, increase expression of BDNF, and promote neurogenesis.	Sooth the liver and relieve depression, invigorate the spleen, and tranquilize the mind.
Chaihu Shugan San	CaiHu (*Bupleurum chinense* DC.), ChenPi (*Citrus reticulata* Blanco), BaiShao (*Paeonia lactiflora* Pall.), ZhiKe (*Citrus aurantium* L.), XiangFu (*Cyperus rotundus* L.), ChuanXiong (*Ligusticum chuanxiong* Hort.), GanCao (*Glycyrrhiza uralensis* Fisch.).	Increase expression of BDNF, neuroprotective effects, and anti-inflammatory.	Dredge the liver to regulate Qi, remove stasis, promote blood circulation, and pain relief.
Ganmai Dazao decoction	GanCao (*Glycyrrhiza uralensis* Fisch.), XiaoMai (*Triticum aestivum* L.), DaZao (*Ziziphus jujube* Mill.)	Increase the activity and level 5-HT and NE, regulate HPA axis elevation, and neuroprotective effects to reduce stress impairments to synapses.	Nourish the heart and tranquilize the mind, harmonize the function of the middle jiao, and alleviate pain.

5-HT, serotonin; DA, dopamine; NE, norepinephrine; HPA, hypothalamic-pituitary-adrenal; BDNF, brain-derived neurotrophic factor.

## CHM for MDD with physical comorbidity

In 2013, MDD was the second highest contributor to global disease burden. Moreover, the consequences of MDD could further lead to impairments in physical health. Large-scale longitudinal studies have suggested that MDD increases the risk of diabetes, heart disease, stroke, hypertension, obesity, cancer, cognitive impairment, and Alzheimer's disease (43). In particular, the cumulative incidence for circulatory conditions after the diagnosis of mood disorders is 40.9%, the highest for any mental disorder (44). However, currently available antidepressants are associated with several safety issues, such as increased bleeding risk, hyponatremia, decreased bone density, and falls, which could become more common and severe in elderly individuals with MDD (45). Therefore, alternative treatments with less adverse effects could represent a solution. In this section, we discuss the effects of CHM treatments on depression comorbid with physical diseases, in a disease-specific manner.

### Depression and coronary heart disease

Numerous pieces of evidence have suggested a high prevalence of major depression or depressive episodes in individuals with cardiovascular heart disease (19). Depression is believed to be a risk factor for cardiovascular heart disease (46). Current treatments for depression in patients with cardiovascular heart disease mainly include antidepressants, cognitive behavioral therapy, interpersonal psychotherapy, and exercise. Most trials have found the interventions to be superior to placebos, with modest effect sizes however. Besides, monoamine oxidase inhibitors and tricyclic antidepressants have cardiotoxic adverse effects, and are therefore seldomly used to treat depression in patients with cardiac diseases (47).

In 2016, Liu et al. conducted an RCT comparing the clinical efficacy and safety of Shugan Jieyu capsules with sertraline in patients with acute myocardial infarction and depression. The results indicated that no significant differences were found in the effectiveness rates between the two interventions, but there was a lower adverse event rate in the Shugan Jieyu treatment group (48). Interestingly, one RCT study on Xinkeshu tablets, previously used as a treatment for coronary heart disease, showed beneficial effects on depressive or anxiety symptoms compared with placebos. These improvements were associated with changes in peripheral blood cytokines, indicating inflammation as the possible mechanistic pathway driving its effects (49). One meta-analysis involving 13 RCT trials and 1,095 participants suggested that a 4-week treatment of CHM showed no difference in reducing the degree of depression, measured with the HAMD, compared with antidepressants. Meanwhile, an 8-week treatment of CHM combined with antidepressants showed a significant advantage in reducing HAMD scores compared with antidepressant monotherapy alone, and was associated with fewer side effects such as thirst, dizziness, and loss of appetite. However, the authors emphasized a careful interpretation of this result due to a high risk of bias (50). Another meta-analysis of 16 RCT studies and 1,443 participants reported a similar result, favoring CHM treatment for depressive disorder in patients after percutaneous coronary intervention (PCI). Compared with antidepressants alone, CHM showed similar benefits with fewer side effects. The combination of CHMs and antidepressants provided more advantages than antidepressants alone. When compared with placebo, it was more effective in relieving depressive symptoms. Furthermore, CHM was effective in relieving chest pain and other general clinical symptoms in terms of post-PCI related symptoms (51).

### Post-stroke depression

Stroke is defined as a sudden loss of blood to the brain causing permanent tissue damage by thrombotic, embolic, or hemorrhagic events. In the most recent meta-analysis including 25,488 patients, Hackett and Pickles reported that 31% of patients developed depression after stroke over a 5-year period (52). Other researches have indicated that being female (53), a personal history of depression or anxiety, and the severity of the disability (54) are possible risk factors for developing post-stroke depression. Vice versa, depression has been shown to be an independent predictor of the severity of the impairment in daily living activities and of higher mortality rates (OR = 3.4, 95% CI = 1.4–8.4, *P* = 0.007) (55). A meta-analysis including 1,655 patients suggested that antidepressants provided significant beneficial effects while psychotherapy was not more effective than a control intervention in patients with depression after stroke (56). However, antidepressants could cause severe side effects as well. For example, SSRI use is associated with an increased risk of hemorrhagic complications and risk of falls in the elderly (57). Two epidemiological studies provide evidence for an increased risk of stroke, myocardial infarction, and all-cause mortality after SSRI treatment (58, 59).

Xiaoyao formula is one of the most commonly used Chinese herbal formulas for treating depression. In 2018, Jin et al. conducted a meta-analysis on the efficacy and safety of Xiaoyao formula as an add-on treatment for post-stroke depression. Seven RCTs and 607 participants with a diagnosis of depression following stroke were included. All the included trials were published in Chinese. The combination of Xiaoyao formula and antidepressants showed an advantage in response rate in HAMD scores compared with antidepressants alone (RR = 1.21, 95% CI = 1.12–1.30) and no significant difference was found on adverse events (60), suggesting its potential use as an add-on treatment.

For Shugan Jieyu capsule, Yao et al. conducted clinical research involving 15 post-stroke depression patients that indicated that it could significantly reduce the depressive symptoms assessed by the HAMD-24 and improve cognitive functions as assessed by the Montreal Cognitive Assessment (MoCA) following 8 weeks of treatment. Furthermore, the decreased dynamic amplitude of low-frequency fluctuation (dALFF) in the right precuneus and increased dynamic functional connectivity between the right precuneus and left angular gyrus were reversed after the intervention. The dALFF variance in the right precuneus was positively correlated with the MoCA score in post-stroke depression patients. These findings suggested a potential treatment indication and the neurobiological mechanisms of Shugan Jieyu for ameliorating symptoms of post-stroke depression (61).

Medication with liver soothing-oriented CHM showed significant benefits as adjunctive treatments in depression after cerebrovascular accidents. One meta-analysis including 30 RCTs and 2,599 patients with depression after cerebrovascular accident suggested that adjunctive treatment with CHM significantly improved the total effectiveness rate and HAMD score in terms of depression symptoms (62), which is similar to the result of treating depression alone.

### Postpartum depression and perimenopausal depression

Postpartum depression, including sleep disturbance, anxiety, irritability, and a feeling of being overwhelmed, is one of the most common complications of childbearing (63). The worst potential consequences include suicide and causing harm to the baby (64). The estimated prevalence of postpartum depression ranges from 6.5% to 12.9% and could be even higher in lower-income and middle-income countries (65). The rapid decline in the level of reproductive hormones after childbirth probably contributes to the development of depression, however, the specific pathogenesis of postpartum depression remains unknown (66). For women with mild symptoms, psychosocial interventions are considered first-line treatment, as they could effectively decrease the likelihood of remaining depressed at 1-year postpartum (67). Besides, formal psychotherapy focusing on the challenges of the transition to parenthood is recommended if the illness is moderate or not responding to psychosocial interventions alone (68). In addition, antidepressant medication is recommended when psychological treatment alone fails to resolve the symptoms of the postpartum depression, when rapid treatment is required, or when the symptoms are severe (67). Most SSRIs pass into breast milk at a proportion of <10% of the maternal dose, and are considered compatible with breastfeeding (69). However, data on long-term child development are very limited. Other possible treatments include hormonal interventions but the clinical evidence is still controversial (70).

Perimenopause is the transitional phase to non-reproductive life that, like the postpartum period, leads to fluctuating hormones. Although the majority of women do not experience negative mood consequences during the menopausal transition, the risk of depressive episodes during the perimenopausal period is much higher than in the premenopausal stage (71). SSRIs and SNRIs are considered to be the first-line treatment for perimenopausal depression, although the delayed onset of efficacy, low remission rates, and side effects limit their application (72). In 2019, Cao et al. conducted an RCT involving 307 participants to evaluate the effectiveness and safety of CHM on perimenopausal depression. The participants were randomly divided into two treatment groups: the Bushen Tiaogan formula plus psychotherapy and placebo plus psychotherapy groups. Bushen Tiaogan was modified from Yi Guan Decoction and consists of eight herbs, as previously described. The results suggested that depressive symptoms were significantly improved in the Bushen Tiaogan formula plus psychotherapy group compared to the placebo plus psychotherapy group, with no serious adverse events occurring (73). One meta-analysis also supported the antidepressant effect of CHMs in menopausal depression. However, all included trials were conducted in China, and the risk of bias was considered high (74).

### Diabetes and depression

Diabetes is currently in seventh place in terms of producing disability, while depression has come up to second place among all diseases worldwide (75). The association of depression and diabetes has been widely reported. For example, the prevalence of depressive disorders in diabetes is about 10–15%, twice as it is in non-diabetic population (76). Moreover, comorbidity could significantly worsen the prognosis of both diseases and increase mortality (77). However, some of the first-line antidepressants could affect blood glucose and aggravate hyperglycemic states (78). Currently, there are few studies published in English focused on the treatment effect and safety of CHMs on diabetes comorbid with depression. Wang et al. conducted an RCT on Wuling capsule, a compound CHM isolated from natural Radix Anophylla, evaluating its effect and safety on type 2 diabetes complicated by depression. Sixty-six patients were enrolled and randomized to either the Wuling or placebo groups and received the treatment for 12 weeks. After the intervention, the HAMD scores were decreased in both groups while the scores in the Wuling capsule group were significantly lower. Meanwhile, the fasting glucose, interleukin-6, and tumor necrosis factor alpha levels were decreased in both groups (79). One meta-analysis including 12 RCTs of 822 diabetes patients with depression indicated that Gardenia Fructus antidepressant formula could significantly reduce the HAMD score compared with no antidepressant treatment. However, due to the high risk of bias in this research, a careful interpretation of the result is necessary (80).

## Possible mechanisms of CHM using modern theories

### Monoamine hypothesis

As mentioned above, the monoamine hypothesis was developed in the mid-twentieth century when reserpine, an anti-hypertensive medication, was found to trigger major depression and reduce the amount of monoamines, which raised the possibility of the potential role of monoamine neurotransmitters in the pathogenesis of major depression. This hypothesis was further supported by the findings that tricyclic antidepressants and monoamine oxidase inhibitors could enhance monoamine neurotransmission and relieve depression (3). This model also endured due to the findings that more selective medications are clinically effective antidepressants (81). *Polygala tenuifolia* Willd is a common component in several empirical formulas for depression treatment, such as Kai Xin San. A triterpenoid saponin isolated from *Polygala tenuifolia* Willd has recently been identified to have a mechanism of action in its antidepressive effect as a triple monoamine reuptake inhibitor with high potency (82). The polysaccharides from Banxia Houpo decoction can significantly increase monoamine neurotransmitter serotonin (5-HT) and dopamine (DA) levels in the whole mouse brain and relive depressive-like behaviors (83). DanZhi Xiaoyao San has also been shown to increase 5-HT and DA levels in the hippocampus in a rat model of depression (84). Baisong tablets and the herb Centella Asiatica also increased brain monoamine neurotransmitter levels in an animal model of depression and exhibited antidepressant effects (85, 86).

### Hypothalamic-pituitary-adrenal axis

For more than a decade, studies have been focusing on the relationship between the HPA axis and depression (87). One popular hypothesis argues that stress leads to activation of the HPA axis that increases the levels of glucocorticoid, which subsequently impairs neuronal survival and neurogenesis and consequently causes depression (88). As mentioned above, DanZhi Xiaoyao San significantly decreased the corticosterone (CORT), corticotropin-releasing hormone (CRH), and adrenocorticotropic hormone (ACTH) levels in the plasma and hypothalamus, indicating that its antidepressive effects are related to the inhibition of a hyperactive HPA axis (84). Shuyu san, a Chinese herbal medication for depression, could also decrease glucocorticoid levels, regulate the function of the HPA axis, and inhibit glucocorticoid receptor expression in the hippocampus (89). The active component of the herb *Polygala tenuifolia*, 3,60-disinapoyl sucrose, improves the abnormality in the HPA axis by reducing the CORT, ACTH, and CRH levels and by enhancing expression of glucocorticoid receptors, thereby relieving depressive-like behavior in chronic mildly stressed rats (90). Oral administration of the Chinese herbal medicine Xiangsu powder significantly suppressed the hyperactivity of the HPA axis in an animal model of depression and improved depressive-like behaviors, indicating the HPA axis as the pathway of its mechanism for the antidepressive effect (91). Xu et al. reported that Ginsenoside Rg3, a protopanaxadiol ginsenoside from *Panax ginseng* C. A. Ney., exerts antidepressive effects through regulating the HPA axis by reducing CRH, CORT, and ACTH in chronic unpredictably stressed animal models (92).

### Inflammation

The immune system, including the cytokine network and inflammatory response, is related to the pathophysiology of depression. For example, peripheral cytokine concentrations have been linked to brain function, wellbeing, and cognition (93). Individuals with MDD show increased activation of the inflammatory system, which is under the inhibitory physiological control of cortisol. Indeed, the immune system, as an important component of the physiological stress-sensing and reactive pathways, may also have a role in MDD. Several animal studies have supported the notion that peripheral immune dysfunction and neuroimmunology are involved in the mechanisms of MDD (8). This hypothesis is further supported by the finding that a higher level of interleukin-6 during childhood is related to a higher risk of depression in later life. Further, a post-mortem study found elevated microglial activation and neuroinflammation in the brains of patients with depression (94). Liu et al. reported that the antioxidant status and anti-inflammatory effects of Icariin, a flavonoid extraction from traditional Chinese herbal Epimedii, could be the possible mechanism of its antidepressive effect (95). Banxia Huopo could relieve depressive behavior in animal models by raising the natural killer (NK) cell activity and interleukin-2 levels (96). In addition, Shenqi Jieyu formula could regulate the immune system and T lymphocyte subsets in a rat model of postpartum depression and improve depressive behaviors (97). Another popular herbal medication, St. John's wort (*Hypericum perforatum*), was reported to inhibit the activation of NK-κB and exert antidepressant effects (98).

### Neuroplasticity and neurogenesis

Growth and adaptability at a neuronal level are broadly termed neuroplasticity and are influenced by inflammation and HPA axis dysfunction due to environmental stress (99). Neurogenesis regulatory proteins, such as brain-derived neurotrophic factor (BDNF), are diminished in patients with depression and could be subsequently restored by either antidepressant therapies or psychological interventions (100). Therefore, neurogenesis has been suggested to facilitate resilience against stress, which could be the basis of the clinical effects of antidepressants (101). A similar hypothesis has been discussed when it comes to the antidepressive effect of traditional Chinese medicine. The antidepressive effect of Shuyu Ningxin has been associated with the increased expression of hippocampal BDNF and its receptor TrkB (102). Shuyu Formula, KaiXin San, and XiaoYao San could also upregulate BDNF in specific brain regions (103–105). Recent studies have demonstrated that chronic administration of ginsenoside Rg1, a protopanaxatriol type of ginsenoside, improves abnormal behavior and increases the phosphorylation level of cAMP-response element binding protein (CREB) and BDNF expression in the prefrontal cortex, which was reduced by chronic unpredictable mild stress modeling (106, 107).

## Discussion

We summarized the currently available and latest evidence for CHM efficacy in the treatment of depression and physiological diseases comorbid with depression. In general, there is some evidence that suggests that CHMs are more effective than placebos and as effective as some first-line antidepressants, with potentially fewer side effects when either used alone or in combination. When considering the accessibility, costs, and preferences of the patients, CHM could be a promising alternative or complementary therapy for MDD or depressive symptoms. However, as some CHM critics have claimed, the number of relevant clinical trials is limited and the present studies may be subject to many shortcomings and publication bias, especially for comorbidity with physical conditions, long-term use side effects, and the efficacy for preventing relapse. Hence, further clinical studies with improved methodology, strict designs, and more comprehensive and better quality are necessary. Besides, from a modern pharmacological theory point of view, most current clinical trials of CHMs on depression did not explore the pharmacokinetic changes of the effective constituent in the human body, and the anti-depressant mechanisms of CHMs have not been well-studied, both of which may be important future research directions. Combinations of Chinese traditional and modern medicine theories may help to better explore the underling mechanisms.

We recommend a careful interpretation of the presented results. In clinical practice, when prescribing CHM to patients with depression, clinicians should be cautious to avoid being misled by unexamined beliefs and be aware that CHM is derived from traditional Chinese medicine theory, which means that the theoretical mechanisms or indications may be different from those of modern medical theory; therefore, the CHM should not be considered fully equal to phytomedicines. Lastly, the patients should be properly informed and educated.

## Author contributions

DK conducted the literature search and drafted the manuscript. HD contributed to the conception and the frame of the study. YS, JO, and JZ reviewed and revised the manuscript. All authors contributed to the article and approved the submitted version.
